# Deletion of Orphan G Protein-Coupled Receptor GPR37L1 in Mice Alters Cardiovascular Homeostasis in a Sex-Specific Manner

**DOI:** 10.3389/fphar.2020.600266

**Published:** 2021-02-09

**Authors:** Margaret A. Mouat, Kristy L. Jackson, James L. J. Coleman, Madeleine R. Paterson, Robert M. Graham, Geoffrey A. Head, Nicola J. Smith

**Affiliations:** ^1^ Molecular Pharmacology Laboratory, Victor Chang Cardiac Research Institute, Sydney, NSW, Australia; ^2^ St Vincent’s Clinical School, UNSW Sydney, Sydney, NSW, Australia; ^3^ Molecular Cardiology and Biophysics Division, Victor Chang Cardiac Research Institute, Sydney, NSW, Australia; ^4^ Neuropharmacology Laboratory, Baker Heart and Diabetes Institute, Melbourne, VIC, Australia; ^5^ Drug Discovery Biology, Monash Institute of Pharmaceutical Sciences, Monash University, Parkville, VIC, Australia

**Keywords:** G protein-coupled receptor, sex differences, hypertension, blood pressure, radiotelemetry, heart rate variability

## Abstract

GPR37L1 is a family A orphan G protein-coupled receptor (GPCR) with a putative role in blood pressure regulation and cardioprotection. In mice, genetic ablation of *Gpr37l1* causes sex-dependent effects; female mice lacking *Gpr37l1* (GPR37L1^−/−^) have a modest but significant elevation in blood pressure, while male GPR37L1^−/−^ mice are more susceptible to cardiovascular dysfunction following angiotensin II-induced hypertension. Given that this receptor is highly expressed in the brain, we hypothesize that the cardiovascular phenotype of GPR37L1^−/−^ mice is due to changes in autonomic regulation of blood pressure and heart rate. To investigate this, radiotelemetry was employed to characterize baseline cardiovascular variables in GPR37L1^−/−^ mice of both sexes compared to wildtype controls, followed by power spectral analysis to quantify short-term fluctuations in blood pressure and heart rate attributable to alterations in autonomic homeostatic mechanisms. Additionally, pharmacological ganglionic blockade was performed to determine vasomotor tone, and environmental stress tests were used to assess whether cardiovascular reactivity was altered in GPR37L1^−/−^ mice. We observed that mean arterial pressure was significantly lower in female GPR37L1^−/−^ mice compared to wildtype counterparts, but was unchanged in male GPR37L1^−/−^ mice. GPR37L1^−/−^ genotype had a statistically significant positive chronotropic effect on heart rate across both sexes when analyzed by two-way ANOVA. Power spectral analysis of these data revealed a reduction in power in the heart rate spectrum between 0.5 and 3 Hz in female GPR37L1^−/−^ mice during the diurnal active period, which indicates that GPR37L1^−/−^ mice may have impaired cardiac vagal drive. GPR37L1^−/−^ mice of both sexes also exhibited attenuated depressor responses to ganglionic blockade with pentolinium, indicating that GPR37L1 is involved in maintaining sympathetic vasomotor tone. Interestingly, when these mice were subjected to aversive and appetitive behavioral stressors, the female GPR37L1^−/−^ mice exhibited an attenuation of cardiovascular reactivity to aversive, but not appetitive, environmental stimuli. Together, these results suggest that loss of GPR37L1 affects autonomic maintenance of blood pressure, giving rise to sex-specific cardiovascular changes in GPR37L1^−/−^ mice.

## Introduction

Over 100 of the ∼800 known human G protein-coupled receptors (GPCRs) (Fredriksson et al., 2003) lack a pairing to an endogenous ligand and are collectively known as ‘orphan’ receptors (Davenport et al., 2013). Orphan GPCRs remain largely understudied (So et al., 2020), owing mainly to the paucity of pharmacological tools available to probe receptor signaling. Many orphan receptors lack an adequate signaling assay, which is a significant roadblock to ligand discovery, and a further hindrance to identification of the physiological role of these orphan GPCRs in health and disease.

GPR37L1 is one such orphan receptor, initially described as a family A GPCR with high sequence homology to another orphan GPCR, GPR37, and to endothelin receptors (Valdenaire et al., 1998). Though there have been attempts to discover the natural ligand for this receptor, there is presently no conclusive evidence for GPR37L1 ‘deorphanization’ and it remains an orphan receptor by IUPHAR classification (Davenport et al., 2013).

Expression of GPR37L1 is largely restricted to the central nervous system, where it is expressed specifically in glia (Valdenaire et al., 1998; Jolly et al., 2018). Though diffusely present throughout the brain, GPR37L1 is most highly expressed in the cerebellum within the molecular and Purkinje cell layers (Valdenaire et al., 1998; Lein et al., 2007). The physiological role for this receptor has not been thoroughly investigated, though it has been reported to play a role in brain development (Marazziti et al., 2013), seizure susceptibility (Giddens et al., 2017), neuroprotection during ischemia (Jolly et al., 2018), and medulloblastoma development (Di Pietro et al., 2019), which appear to be related to its expression within the central nervous system.

In addition to these effects in the brain, GPR37L1 has a reported role in maintaining blood pressure homeostasis (Min et al., 2010; Coleman et al., 2018; Zheng et al., 2018), and may represent a potential drug target for the treatment of hypertension (Smith, 2015). Supporting this, the closest non-orphan relative of GPR37L1 by phylogeny is the endothelin B receptor (Valdenaire et al., 1998), which is known to have cardiovascular effects (Mouat et al., 2018). In addition, GPR37L1 itself is encoded within a renin blood pressure quantitative trait locus, specifically in a sub-region that promotes high blood pressure in rats (Flister et al., 2013). Indeed, there is evidence that genetic deletion of *Gpr37l1* in mice may cause high blood pressure (Min et al., 2010). We have previously found that the cardiovascular effects of *Gpr37l1* deletion are sexually dimorphic, with female GPR37L1 knockout (GPR37L1^−/−^) mice exhibiting higher blood pressure than wildtype controls (Coleman et al., 2018). In contrast, male GPR37L1^−/−^ mice developed greater left ventricular hypertrophy and evidence of heart failure in response to short-term angiotensin II infusion, suggesting that this receptor has a sex-specific role in maintaining blood pressure homeostasis and in responding to pathological conditions (Coleman et al., 2018).

However, the mechanism by which GPR37L1 exerts its effects on blood pressure is not currently understood. It has been suggested that GPR37L1 may influence blood pressure by maintaining renal sodium reabsorption (Zheng et al., 2018), though GPR37L1 protein is undetectable in mouse kidney using a knockout tissue-validated GPR37L1 antibody (Coleman et al., 2018). There is also no evidence of expression of GPR37L1 protein in the mouse heart (Coleman et al., 2018), and it is therefore unlikely that GPR37L1 mediates cardiovascular homeostasis through local activity in cardiovascular effector organs such as the kidney or heart. Rather, based on the finding that GPR37L1 protein expression is largely restricted to the brain in mice and in humans (Uhlen et al., 2015; Smith et al., 2019), we propose that it influences autonomic cardiovascular regulation via a central mechanism that alters sympathetic nerve activity.

Our aim here was to determine whether GPR37L1 influences cardiovascular homeostasis via modulation of the autonomic nervous system. Specifically, we investigated the effects of murine *Gpr37l1* ablation on cardiovascular fluctuations associated with autonomic activity, sympathetic vasomotor tone and response to aversive and appetitive behavioral stressors.

## Materials and Methods

### Generation and Maintenance of GPR37L1 Knockout Mouse Line

Male and female *Gpr37l1* knockout mice on a C57BL/6J background (hereafter referred to as “GPR37L1^−/−^”) were generated at the Victor Chang Cardiac Research Institute using the European Conditional Mouse Mutagenesis Program (EUCOMM) method for conditional gene inactivation using *Cre/loxP* and *Flp1/FRT* gene-trapping (Skarnes et al., 2011) described as GOI#1 in Coleman et al. (2015). Experimental mice were produced from homozygous breeding pairs that were no further than five generations from a heterozygous mating to minimize genetic drift between C57BL/6J and GPR37L1^−/−^ lines.

### Ethics Statement and Animal Husbandry

All animal work was performed at the Baker Heart and Diabetes Institute with appropriate ethics approval (approved by the Alfred Medical Research and Education Precinct Animal Ethics Committee, project number E/1793/2018/B) and in accordance with the Australia Code for the Care and Use of Animals for Scientific Purposes, 8th Edition (2013). All animal breeding, transportation and experimentation was conducted in accordance with the Commonwealth of Australia Gene Technology Act 2000 and Gene Technology Regulations 2001 and approved by the relevant Institutional Biosafety Committees (notifiable low risk dealings N80, N143 and 11974). Experimental procedures were performed in accordance with locally approved safe work procedures and risk assessments. Mice were housed on a 12-h light cycle (lights on 0100 h), with standard chow (Specialty Feeds, Australia; 19% protein, 5% fat, 5% fiber, 0.2% sodium) and water available *ad libitum* (except for temporary fasting prior to feeding behavioral tests). Mice were group-housed prior to radiotelemeter implantation, then individually for the remaining experimental program.

### Radiotelemetry Blood Pressure and Locomotor Activity Measurement

At 12–14 weeks of age mice were anesthetized (induction: 3.5% isoflurane; maintenance: 1.5% isoflurane, both in O_2_ at 200 ml/min; Forthane, Abbott, Botany, NSW, Australia) and a blood pressure (BP) telemetry transmitter (model TA11PA-C10; Data Sciences International, USA) implanted subcutaneously with the catheter inserted into the left carotid artery and the pressure-sensing probe body placed subcutaneously on the right flank, as previously described (Jackson et al., 2013).

Prior to surgery, animals received SC carprofen (5 mg/kg; Rimadyl, Pfizer Australia), IP atropine (1.2 mg/kg, Sigma, St Louis, Missouri, United States), IP Hartmann’s solution (0.3 ml) and intradermal bupivacaine (2 mg/kg, AstraZeneca, North Ryde, NSW, Australia) at the incision site. Mice were allowed to recover for 10 days following telemetry implantation surgery to allow return of normal circadian blood pressure fluctuations (Butz and Davisson, 2001). After this recovery period, radiotelemeters were magnetically activated and allowed to record baseline (minimum 60 h) BP, heart rate (HR), respiration rate and locomotor activity while mice remained in their home cage. Parameters were sampled at 1,000 Hz using an analog-to-digital data acquisition card (National Instruments 6024E) as described previously (Jackson et al., 2007). Data was stored as beat-to-beat average, 2 s averaged, and minute averaged data (SBP, DBP, calculated mean arterial pressure [MAP], HR, activity, respiratory rate) and analyzed off-line using a program written in LabView (National Instruments, United States) (Head et al., 2001).

### MAP-Activity Relationship

To assess the correlation between locomotor activity and MAP levels, 2-s averages of log-locomotor activity were plotted against 2-s averages of MAP for each mouse using a 6-s delay (to account for the temporal relation between variables) for a 12-h period taken from baseline radiotelemetry measurements (Jackson et al., 2016). This 12-h period encompasses 6 h of the dark period followed by 6 h of the light period. Least squares linear regression and correlation coefficient were determined for MAP vs. log-locomotor activity for each animal, and MAP was evaluated at log-locomotor activity equals 0 to give intercept; mean and SEM were determined within each experimental group from these data.

### Power Spectral Analysis

Power spectral analysis was performed on baseline long-term blood pressure readings using a program written in LabView as described previously (Head et al., 2004). Briefly, the fast Fourier transform was used to transform beat-to-beat HR and mean arterial BP data into the frequency domain. Spectral power was calculated within the defined frequency ranges: 0.08–0.3 Hz (low frequency), 0.3–0.5 Hz (mid frequency) and 0.5–3 Hz (high frequency). These frequency ranges have been selected to specifically investigate oscillations that are modulated by autonomic mechanisms; mid frequency MAP oscillations (Mayer waves) reflect sympathetic activity (Just et al., 2000), while high frequency HR oscillations reflect parasympathetic activity (Baudrie et al., 2007).

The data was resampled at 40.96 Hz using the cubic interpolation method. Periods of relative stationarity in the data were selected for by using a running short-term standard deviation (40 points) for each variable, and manually excluding data with high SD. The remaining data periods were then partitioned into segments of 50 s (2048 points) in length, overlapping by 50%, and only data periods comprising more than four consecutive segments were included. The subsequent spectral analysis was performed according to the Welch periodogram method. Each segment was detrended using linear regression, windowed with a tapered cosine function, and padded with zeros up to 2048 points. The resulting frequency step was 0.01 Hz. The auto- and cross-spectral power were calculated for each segment using the fast Fourier transform, and then subjected to ensemble averaging. To quantify how regular fluctuations in MAP influence regular fluctuations in HR, the gain (magnitude) and phase (temporal relationship) of the transfer function between MAP and HR were computed. In addition, coherence (correlation coefficient) was calculated for both auto- and cross-power spectra. Only periods of data where average coherence values were greater than 0.5 in the mid-range frequency (0.3–0.5 Hz) were used in the estimation of baroreflex gain. In both active and inactive periods, four segments were sampled for each animal from the 72-h continuous recording. Active and inactive periods were analyzed separately.

### Pharmacological Autonomic Blockade with Renin-Angiotensin System Pre-Inhibition

At 15–17 weeks of age, blockade of the sympathetic nervous system (SNS) was performed using pentolinium, after pretreatment with the angiotensin-converting enzyme inhibitor, enalaprilat, as previously described (Jackson et al., 2013). Enalaprilat pretreatment was used to block the peripheral renin-angiotensin system (RAS) (enalaprilat does not readily cross the blood-brain barrier (Cohen and Kurz, 1982)), thus serving to abolish blood pressure compensation by the RAS following pentolinium treatment (Gardiner and Bennett, 1985). Subsequent ganglionic blockade with pentolinium reduces sympathetic vasomotor drive, resulting in a rapid fall in blood pressure and heart rate (Davern et al., 2009). During the test, cardiovascular parameters and activity levels were recorded by radiotelemetry during a stable baseline period (30 min), followed by sequential administration of treatments (each followed by a 30-min recording period): vehicle (0.9% saline, Baxter International Inc., IL, United States), enalaprilat (1 mg/kg; Merck and Co., Keniworth, NJ, United States), and pentolinium (5 mg/kg, Sigma-Aldrich, Castle Hill, NSW Australia). This pharmacological autonomic blockade was performed during both the light (inactive) and dark (active) periods of the diurnal cycle, with at least 1 day of recovery between assessments. Statistical analysis was performed on the delta values between plateau periods of each treatment vs the immediately preceding treatment (plateau periods: control *t* = 15–30; vehicle *t* = 45–60; enalaprilat *t* = 75–90; pentolinium *t* = 100–115).

### Physical Stress Tests

Physical stress tests were performed on telemetered mice at 14–17 weeks of age. Tests were all performed during the light period, and on separate days to avoid carry-over effects. A stable 1-h baseline telemetry recording period was obtained prior to each stressor, followed by a 1-h recording for the duration of the test. *Restraint (aversive stimulus)*: mice were restrained in a plexiglass restrainer (Harvard Apparatus, South Natick, MA; length 135 mm, diameter 50 mm), with stopper tightened to restrict movement. *Dirty cage swap (aversive stimulus)*: mice were changed into a soiled cage (previously inhabited by a non-littermate male mouse for at least 1 week), and then transferred into a fresh home cage after test completion. *Feeding (appetitive stimulus)*: mice were fasted for approximately 12 h prior to test, then fed a sliver of almond (Coles loose almonds, Coles, Australia) every 10 min for 1 h.

### Light-Dark Transition Test

Mice were scored for anxiety behaviors using the light-dark transition test at 15–16 weeks of age. The apparatus consisted of two sections, the dark compartment (13 × 29 × 13 cm) covers one third of the apparatus area and is completely enclosed with the exception of a 5 × 5 cm opening into the light compartment (26 × 29 × 13 cm), which spans the remaining two thirds of the apparatus. The light compartment is illuminated by a 50-W light source directly above the apparatus. Mice were placed in the center of the light section at the beginning of the test, and behavior scored for 5 min. Parameters measured: percentage of time spent in the light section (timed), number of transitions (number of times the mouse completely returned to the light section, defined by the front two paws in contact with the section), nose pokes (number of less than complete transitions through passageway), rearing events (number of rearing events in the light section, defined by the two front paws being lifted off the ground). A singular operator performed all testing in a genotype-blinded fashion to minimize bias.

### Statistics and Data Analysis

Histograms show mean ± SEM, with individual data points represented by open circles. XY plots show mean ± SEM. Statistical tests were performed using purpose-built Microsoft Excel spreadsheets, or using in-built analysis in GraphPad Prism version 8.0.2. For experiments with repeated or continuous long-term measurements for each subject, split plot ANOVA was used with Greenhouse-Geisser adjustment for sphericity, with Bonferroni correction for multiple comparisons. For experiments with singular measures of dependent variables, ordinary two-way ANOVA was used, with Bonferroni correction for multiple comparisons. In all cases, significance threshold was defined as *p* < 0.05. *A priori* and *a posteriori* statistical power calculations were performed using G*Power 3.1 (Faul et al., 2007).

## Results

### Radiotelemetry Recording of Baseline Blood Pressure in GPR37L1^−/−^ Mice

**Figure 1:**
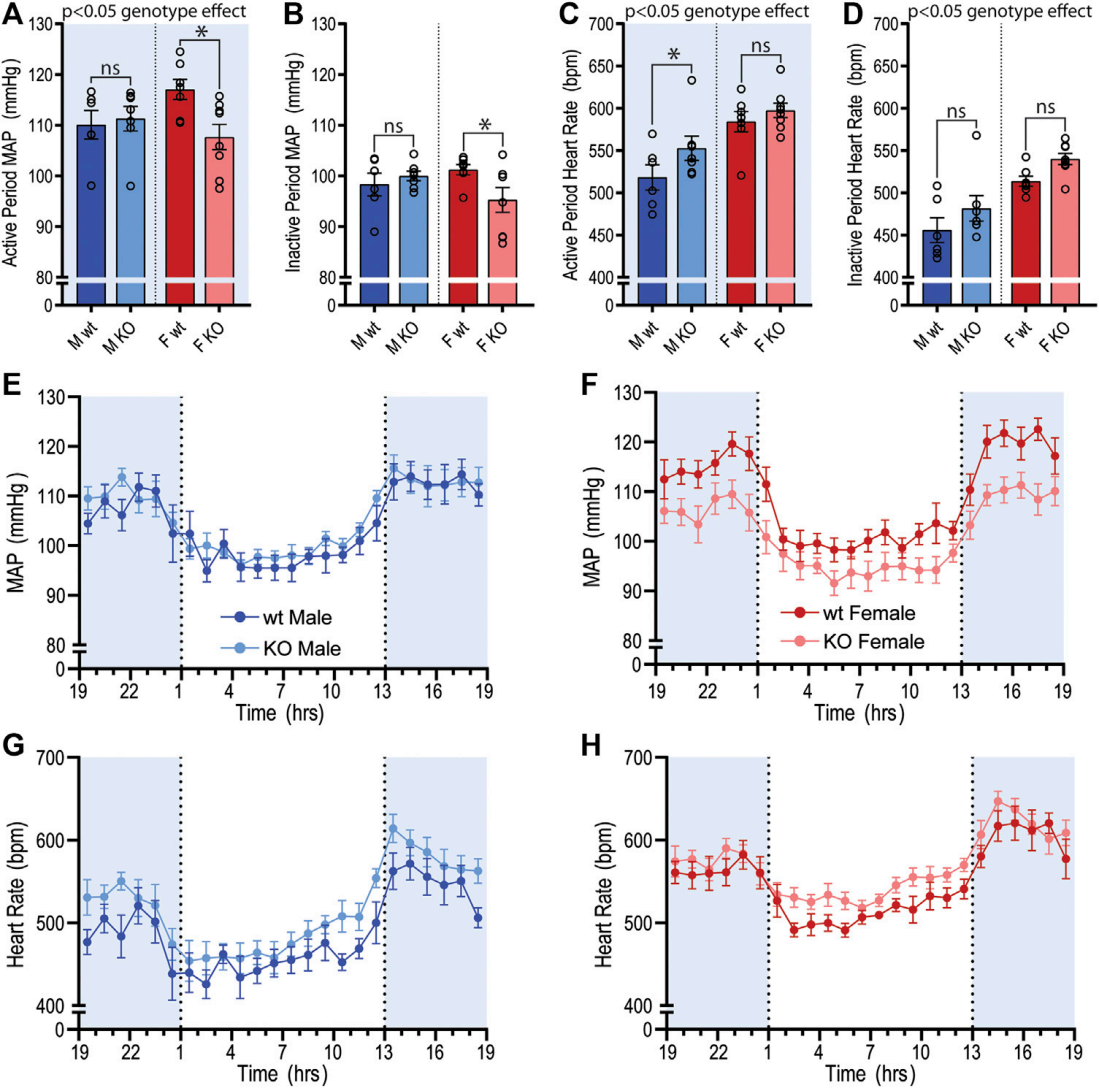
Baseline mean arterial pressure and heart rate recordings using radiotelemetry in GPR37L1^−/−^ mice. Cardiovascular parameters were recorded by radiotelemetry for male and female wildtype (C57BL/6J; wt) and GPR37L1^−/−^ (KO) mice over a continuous period of ≥60 h at 14–15 weeks of age. Average mean arterial pressure (MAP) over the active period (1300–0100h; **(A)**) and inactive period (0100–1300h; **(B)**). Average heart rate over the active period **(C)** and the inactive period **(D)**. For clarity, sexes are plotted separately; plot of hourly average MAP for males **(E)** and females **(F)**, heart rate for males **(G)** and females **(H)**. Males *n* = 6–7, females *n* = 7–8. XY plot data points are mean ± SEM averaged over each hour (average of ≥60 h recording). Bar graphs show mean ± SEM, open circles represent 12 h averages for individual subjects, statistical analysis was performed on hourly averages using split plot ANOVA with Bonferroni correction for multiple comparisons. Shaded areas indicate dark period (lights off 1300 to 0100 h).

The baseline cardiovascular phenotype of 14–15-week-old GPR37L1^−/−^ and wildtype mice of both sexes was evaluated using radiotelemetry, the current gold standard for obtaining high-fidelity, continuous blood pressure and heart rate recordings in conscious mice in their home cage (Zhao et al., 2011). We observed significant genotype differences in SBP, MAP, HR and locomotor activity between GPR37L1^−/−^ and wildtype mice when telemetry data was averaged over the active and inactive periods (Figures 1A–D; Table 1). Importantly, there was a statistically significant interaction between genotype and sex on SBP (interaction effect *p* < 0.001, two-way ANOVA) and MAP (interaction effect *p* < 0.001, two-way ANOVA) over 24 h, indicating that genetic ablation of *Gpr37L1* affects blood pressure in male and female mice differently.

**TABLE 1:** Cardiovascular variables and locomotor activity averaged over a 24-h period in male and female wildtype and GPR37L1^−/−^ mice.

24-h average																											
	WT male		KO male			WT female				KO female			Effect of sex				Effect of genotype		Genotype X sex				Male WT:KO			Female WT:KO	
SBP (mmHg)	119.2 ± 2.9		119.0 ± 2.8			121.8 ± 2.2				113.3 ± 2.8			>0.50				<0.001		<0.001				>0.50			<0.001	
DBP (mmHg)	88.7 ± 2.9		91.4 ± 1.7			95.9 ± 2.5				92.0 ± 5.0			0.13				>0.50		0.33				0.50			0.46	
MAP (mmHg)	104.2 ± 2.7		105.6 ± 2.0			109.1 ± 1.9				101.5 ± 2.6			>0.50				0.01		<0.001				>0.50			<0.001	
HR (bpm)	487.0 ± 17.0		517.1 ± 15.5			548.9 ± 11.4				568.8 ± 9.9			<0.001				<0.001		>0.50				0.25			0.09	
Activity (units)	1.17 ± 0.19		0.82 ± 0.15			1.41 ± 0.30				1.12 ± 0.23			0.04				0.01		>0.50				>0.50			0.28	
Respiration (breaths/min)	156.5 ± 4.7		166.2 ± 6.4			176.8 ± 5.2				174.2 ± 4.0			<0.001				>0.50		0.23				0.02			>0.50	
*n*	6		7			7				8																	

Active period average																											
	WT male		KO male			WT female				KO female			Effect of sex				Effect of genotype		Genotype X sex				Male WT:KO			Female WT:KO	
SBP (mmHg)	125.4 ± 1.8		124.8 ± 2.0			129.9 ± 1.5				119.5 ± 1.5			>0.50				0.003		0.01				>0.50			<0.001	
DBP (mmHg)	94.6 ± 1.8		97.1 ± 1.1			103.6 ± 1.5				98.2 ± 2.7			0.21				>0.50		>0.50				>0.50			>0.50	
MAP (mmHg)	110.1 ± 1.7		111.2 ± 1.5			117.1 ± 1.3				107.7 ± 1.5			>0.50				0.01		0.001				>0.50			<0.001	
HR (bpm)	518.3 ± 12.4		552.6 ± 10.8			584.2 ± 8.6				597.5 ± 7.5			<0.001				0.04		>0.50				0.03			>0.50	
Activity (units)	1.82 ± 0.16		1.35 ± 0.13			2.23 ± 0.23				1.82 ± 0.18			0.01				0.01		>0.50				0.18			0.26	
Respiration (breaths/min)	168.8 ± 3.3		179.4 ± 3.7			190.9 ± 2.8				189.1 ± 2.7			<0.001				>0.50		>0.50				0.17			>0.50	
*n*	6		7			7				8																	

Inactive period average																											
		WT male		KO male				WT female			KO female			Effect of sex			Effect of genotype			Genotype X sex			Male WT:KO				Female WT:KO
SBP (mmHg)		113.1 ± 1.4		113.2 ± 1.1				113.7 ± 0.9			107.1 ± 1.5			>0.50			0.30			0.17			>0.50				0.03
DBP (mmHg)		82.8 ± 1.4		85.7 ± 0.9				88.2 ± 1.2			85.7 ± 2.8			>0.50			>0.50			>0.50			>0.50				>0.50
MAP (mmHg)		98.3 ± 1.3		100.0 ± 0.8				101.2 ± 0.8			95.2 ± 1.4			>0.50			>0.50			0.03			>0.50				0.02
HR (bpm)		455.7 ± 9.0		481.6 ± 10.2				513.7 ± 5.1			540.1 ± 5.7			<0.001			0.01			>0.50			0.25				0.14
Activity (units)		0.52 ± 0.04		0.28 ± 0.03				0.60 ± 0.06			0.43 ± 0.04			>0.50			>0.50			>0.50			>0.50				>0.50
Respiration (breaths/min)		144.2 ± 2.6		153.0 ± 4.1				162.8 ± 3.2			159.4 ± 2.5			0.001			>0.50			>0.50			0.48				>0.50
*n*		6		7				7			8																

Active period average - inactive period average																											
		WT male		KO male			WT female			KO female			Effect of sex			Effect of genotype			Genotype X sex			Male WT:KO		Female WT:KO			
ΔSBP (mmHg)		12.3 ± 1.0		11.6 ± 2.1			16.2 ± 1.4			12.4 ± 0.6			0.23			0.21			>0.50			>0.50		0.12			
ΔDBP (mmHg)		11.8 ± 0.8		11.4 ± 1.9			15.4 ± 1.4			12.5 ± 0.9			0.21			0.42			>0.50			>0.50		0.27			
ΔMAP (mmHg)		11.8 ± 0.9		11.3 ± 2.0			15.8 ± 1.4			12.4 ± 0.6			0.14			0.26			>0.50			>0.50		0.14			
ΔHR (bpm)		62.6 ± 7.8		71.0 ± 4.5			70.5 ± 8.8			57.4 ± 12.9			>0.50			>0.50			>0.50			>0.50		>0.50			
ΔActivity (units)		1.30 ± 0.18		1.07 ± 0.15			1.63 ± 0.30			1.39 ± 0.23			0.33			>0.50			>0.50			>0.50		>0.50			
ΔRespiration (breaths/min)		24.5 ± 1.8		26.4 ± 1.5			28.1 ± 2.8			29.7 ± 3.7			0.46			>0.50			>0.50			>0.50		>0.50			
*n*		6		7			7			8																	

MAP was significantly lower in female GPR37L1^−/−^ mice compared to wildtype counterparts by approximately 9 mmHg during the active period, and approximately 6 mmHg during the inactive period (Figures 1A,B,F; Table 1). Specifically, female GPR37L1^−/−^ mice had significantly lower SBP compared to wildtype females, with no statistical differences in DBP between the genotypes (Table 1). In contrast, male GPR37L1^−/−^ mice did not show statistical difference in MAP compared to sex-matched wildtype when expressed as 12-h averages of the active (dark cycle, 1300–0100 h) and inactive periods (light cycle, 0100–1300 h) (Figures 1A,B,E; Table 1).

GPR37L1^−/−^ genotype had a statistically significant positive chronotropic effect on the HR during both the active (genotype effect *p* = 0.041, two-way ANOVA) and inactive (genotype effect *p* = 0.012, two-way ANOVA) periods across both sexes of mice (Table 1). By individual comparisons, male GPR37L1^−/−^ mice had significantly higher HR than wildtype male mice during the active period, though this difference was not observed during the inactive period (Figures 1C,D,G; Table 1).

**Figure 2:**
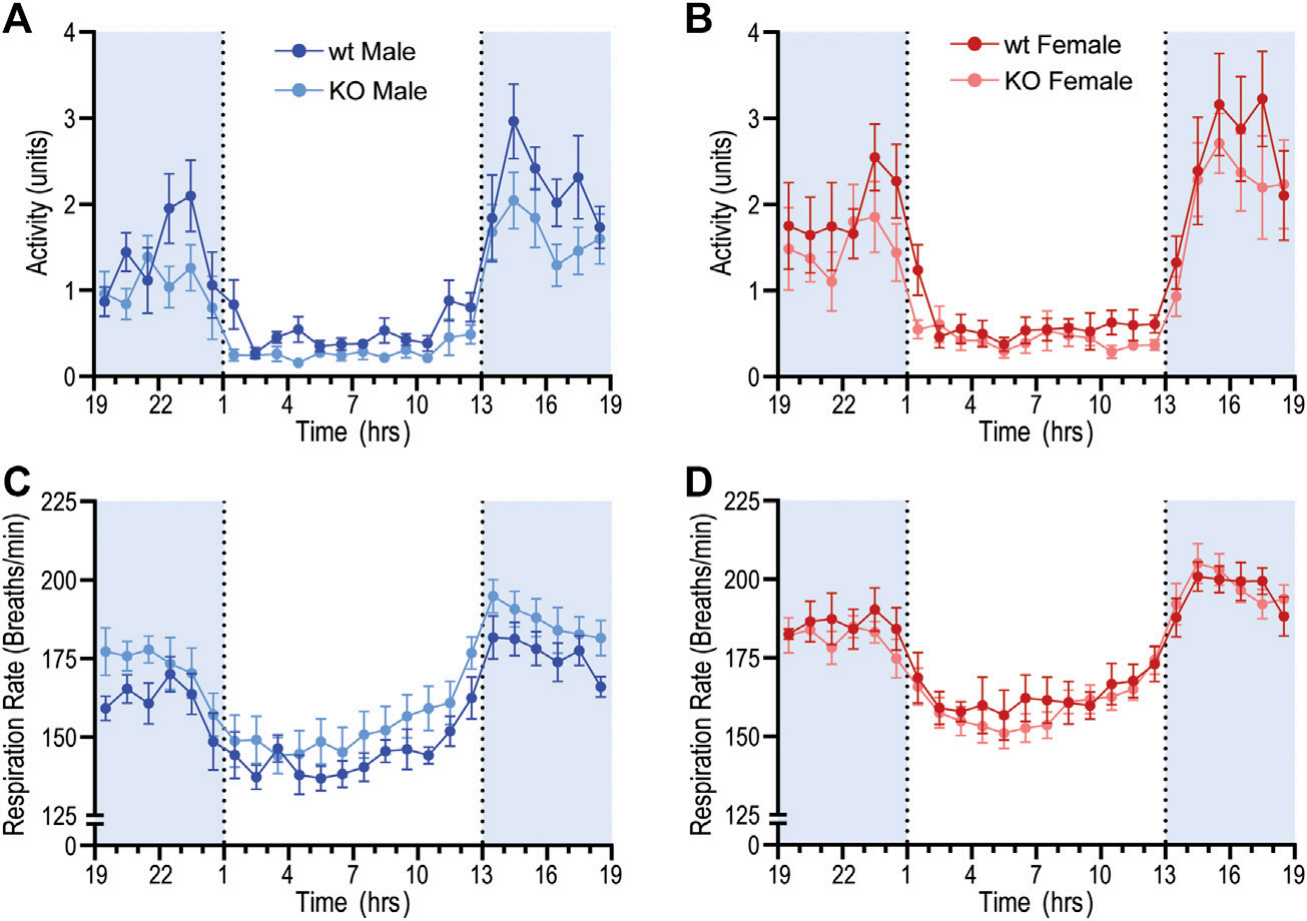
Baseline radiotelemetry recordings for locomotor activity and respiration rate. Locomotor activity and respiratory rate was recorded via radiotelemeter for male and female wildtype (C57BL/6J; wt) and GPR37L1^−/−^ (KO) mice over a continuous period of ≥60 h at 14–15 weeks of age. Locomotor activity (arbitrary units) for males **(A)** and females **(B)**. Respiratory rate for males **(C)** and females **(D)**. Data points are mean ± SEM averaged over each hour (average of ≥60 h recording), males *n* = 6–7, females *n* = 7–8. Shaded areas indicate dark period (lights off 1300 to 0100 h).

GPR37L1^−/−^ genotype again had a statistically significant effect on locomotor activity; across both sexes, locomotor activity was higher in GPR37L1^−/−^ mice compared to wildtype (genotype effect *p* = 0.014, two-way ANOVA). When considering individual multiple comparisons, locomotor activity was not statistically different within each sex when comparing GPR37L1^−/−^ to wildtype mice (Figures 2A,B; Table 1). Imputed respiration rate was statistically higher in female mice compared to male mice (sex effect *p* < 0.001, two-way ANOVA; Table 1; Figures 2C,D). When averaged over 24 h, male GPR37L1^−/−^ mice had significantly higher respiration rate than wildtype males, though this was not observed within the inactive or active periods alone. Respiration rate in female mice was comparable between the genotypes in both active and inactive periods. Of note, the amplitude of circadian fluctuations in MAP, HR, locomotor activity and respiration rate was preserved in GPR37L1^−/−^ mice of both sexes (Figures 1E–H, 2; Table 1).

### MAP-Activity Relationship

**Figure 3:**
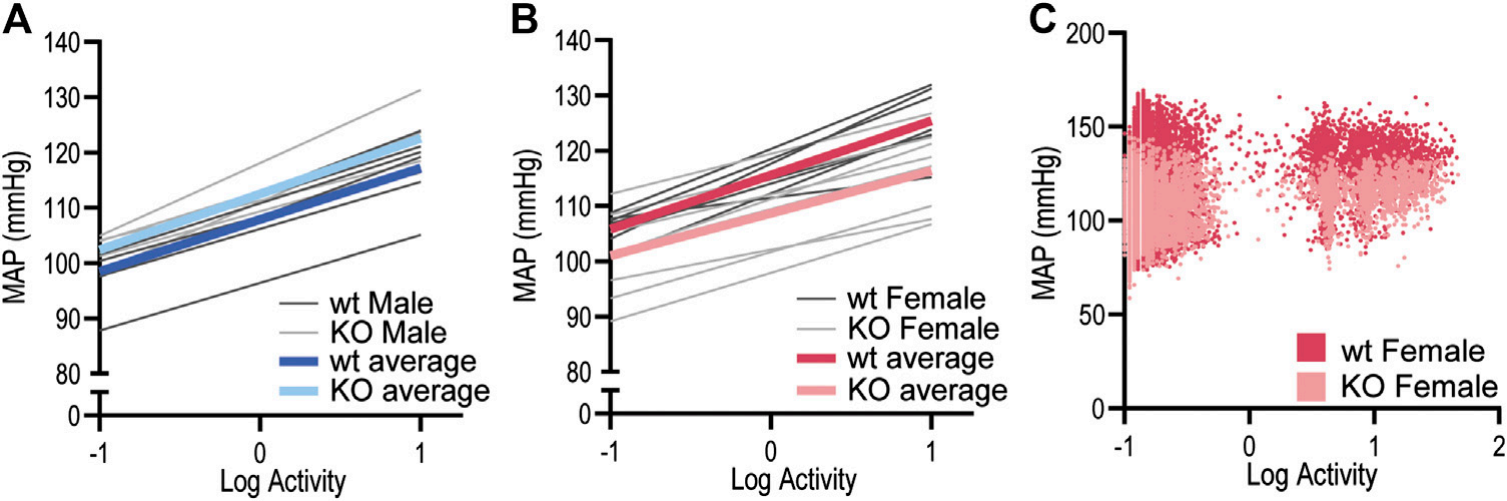
Relationship between MAP and locomotor activity in GPR37L1^−/−^ mice. Linear regressions for mean arterial pressure (MAP) vs. log-locomotor activity in male (*n* = 6–7) **(A)** and female (*n* = 7–8) **(B)** GPR37L1^−/−^ (KO; light gray) and wildtype (wt; C57BL/6J; dark gray) mice during baseline radiotelemetry recordings over a 12-h period (6 h of dark and 6 h of light), with colored lines representing average regression for each genotype. Representative plot of 2-s averages of log-locomotor activity against 6-s-offset MAP over the 12-h period for one wildtype female (darker red) and one GPR37L1^−/−^ female (lighter red) (**C**).

To determine whether the MAP differences observed in GPR37L1^−/−^ mice may be explained by locomotor activity levels, we performed a correlation analysis of MAP vs. log-transformed locomotor activity levels in both sexes of GPR37L1^−/−^ mice compared to wildtype controls. For all subjects, regression slopes were positive, (Figures 3A and 3B) and average regression slopes and correlation coefficients for each experimental group were also positive (Figures 3A and 3B; Table 2), indicating that higher locomotor activity is associated with higher MAP. There were no statistically significant effects of sex or genotype on the regression slopes or correlation coefficients for MAP-activity relationships by two-way ANOVA (Table 2). Representative data (Figure 3C) indicated that there may be genotype differences in the regression offset between genotypes, hence we evaluated and tested for genotype differences in regression line intercepts at log activity equals 0. We observed that GPR37L1^−/−^ female mice had statistically lower MAP compared to wildtype females when log-activity was equal to 0, while there were no significant differences in regression offset between male genotypes. Interestingly, two-way ANOVA indicated a significant interaction effect between sex and genotype for MAP at log-activity equals 0.

**TABLE 2:** Regression slope, correlation and intercept values for MAP-activity relationship.

	WT male	KO male	WT female	KO female	Effect of sex	Effect of genotype	Sex X genotype	Male WT:KO	Female WT:KO
Slope (mmHg/Log activity unit)	9.4 ± 0.4	10.1 ± 1.0	9.8 ± 1.2	7.7 ± 0.5	0.43	>0.50	0.18	>0.50	0.15
MAP at 0 log activity (mmHg)	107.9 ± 2.1	112.5 ± 1.2	115.6 ± 1.3	108.7 ± 2.6	>0.50	>0.50	0.016	0.26	0.039
Correlation coefficient	0.25 ± 0.01	0.31 ± 0.01	0.30 ± 0.04	0.27 ± 0.02	>0.50	>0.50	0.36	0.37	>0.50

### Power Spectral Analysis of Blood Pressure and Heart Rate in GPR37L1^−/−^ Mice

**Figure 4:**
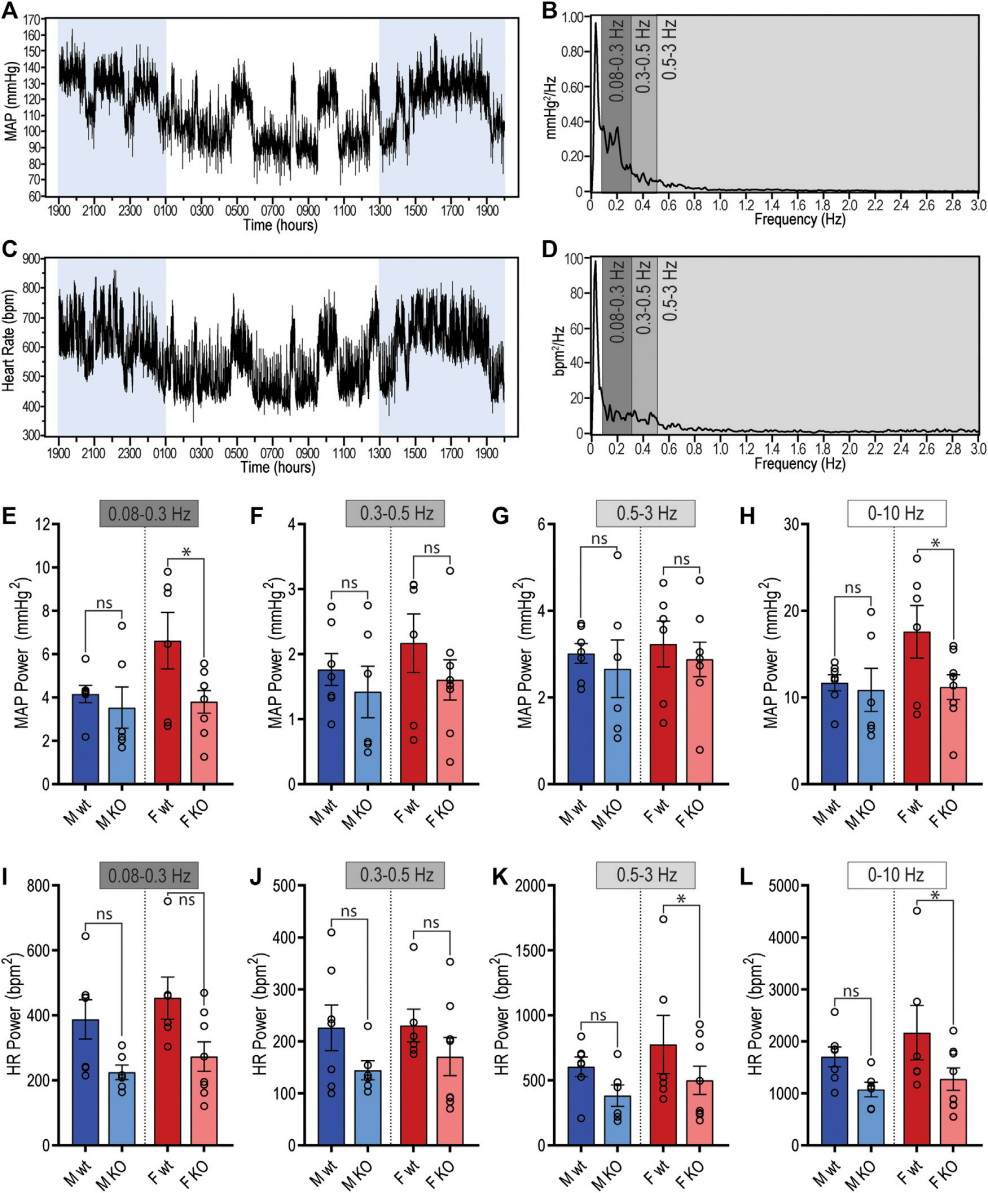
Spectral power analysis of radiotelemetry cardiovascular recordings in GPR37L1^−/−^ (KO) mice. Representative mean arterial pressure (MAP; **(A)**) and heart rate **(C)** recording of a C57BL/6J mouse over a 24-h period (lights on 0100–1300 h). Segments of this recording were subjected to spectral power analysis, with representative power spectra in the frequency domain given for MAP **(B)** and heart rate **(D)**. Cumulative spectral power was calculated in the low (0.08–0.3 Hz), medium (0.3–0.5 Hz), high (0.5–3 Hz) and total (0–10 Hz) frequency bands for both MAP **(E)–(H)** and heart rate **(I)–(L)** radiotelemetry recordings from C57BL/6J wildtype (wt) and GPR37L1^−/−^ (KO) mice during active period at 14–15 weeks of age. Males *n* = 6–7, females *n* = 6–8, analyzed by split plot ANOVA of four spectral sequences with Bonferroni correction for multiple comparisons. Bar graphs represent mean ± SEM; * represents *p* < 0.05 vs. wt, ns is not significant.

To quantify potential changes to autonomic contribution to BP in GPR37L1^−/−^ mice, radiotelemetry recordings were subjected to power spectral analysis to quantify oscillations in BP and HR recordings that are susceptible to autonomic modulation. Recordings of MAP (Figure 4A) and heart rate (Figure 4C) from a singular animal reveal the correlative trends between MAP and HR over 24 h. Representative power spectra for MAP (Figure 4B) and HR (Figure 4D) from the same animal show the expected peak in power below 0.08 Hz, encompassing all very-low frequency rhythmic changes in MAP and HR (including circadian differences), with minimal fluctuations in MAP and HR seen above 0.8 Hz.

In male mice, there were no genotype differences in the power of either MAP or HR spectra within any of the specified frequency bands during the active period (1300–0100 h) (Figures 4E–L; Table 3). During the inactive period, male GPR37L1^−/−^ mice had significantly lower power within the low and mid frequency bands of the HR power spectra (Table 3).

**TABLE 3:** Auto- and cross-spectral parameters during the night (active) and day (inactive) periods.

**Active period**				
	WT male	KO male	WT female	KO female
MAP (mmHg)	109.9 ± 1.3	111.8 ± 2.1	115.5 ± 3.0	106.9 ± 1.9*
HR (bpm)	512.9 ± 14.4	532.7 ± 19.8	567.5 ± 25.1	569.1 ± 15.0
MAP power (mmHg^2^)				
Low frequency (0.08–0.3 Hz)	4.16 ± 0.45	3.53 ± 0.49	6.61 ± 0.95	3.80 ± 0.45*
Mid frequency (0.3–0.5 Hz)	1.76 ± 0.28	1.42 ± 0.22	2.17 ± 0.34	1.60 ± 0.23
High frequency (0.5–3 Hz)	3.01 ± 0.41	2.66 ± 0.35	3.23 ± 0.45	2.88 ± 0.33
Total	11.69 ± 1.27	10.87 ± 1.28	17.59 ± 2.56	11.19 ± 1.22*
HR power (bpm^2^)				
Low frequency (0.08–0.3 Hz)	387.3 ± 59.0	224.7 ± 22.4	453.2 ± 51.2	272.9 ± 32.7
Mid frequency (0.3–0.5 Hz)	226.1 ± 44.2	144.3 ± 15.5	230.3 ± 26.9	170.5 ± 22.5
High frequency (0.5–3 Hz)	604.4 ± 63.1	381.8 ± 53.3	776.2 ± 155.0	498.8 ± 61.9*
Total	1705 ± 176	1,074 ± 100	2,166 ± 341	1,274 ± 129*
Cross-spectral parameters (0.3-0.5 Hz)				
Coherence	0.57 ± 0.01	0.61 ± 0.02	0.59 ± 0.02	0.55 ± 0.01
Gain (bpm/mmHg)	9.19 ± 0.61	9.29 ± 0.71	9.51 ± 0.78	9.55 ± 0.97
Phase (pi fraction)	0.09 ± 0.11	−0.08 ± 0.19	0.24 ± 0.12	−0.26 ± 0.22

Inactive period				
	WT male	KO male	WT female	KO female
MAP (mmHg)	100.8 ± 2.2	101.0 ± 2.0	107.7 ± 2.4	97.0 ± 1.5*
HR (bpm)	465.5 ± 18.2	460.8 ± 15.0	513.2 ± 15.8	513.3 ± 13.0
MAP power (mmHg^2^)				
Low frequency (0.08–0.3 Hz)	4.10 ± 0.45	3.09 ± 0.42	4.37 ± 0.43	3.39 ± 0.34
Mid frequency (0.3–0.5 Hz)	1.56 ± 0.31	0.93 ± 0.19	1.78 ± 0.27	1.66 ± 0.28
High frequency (0.5–3 Hz)	2.30 ± 0.38	1.83 ± 0.39	2.91 ± 0.39	2.71 ± 0.39
Total1	0.95 ± 1.27	8.73 ± 1.13	12.20 ± 1.23	9.92 ± 0.96
HR power (bpm^2^)				
Low frequency (0.08–0.3 Hz)	661.2 ± 156.1	305.3 ± 44.8*	470.7 ± 63.6	265.5 ± 29.3
Mid frequency (0.3–0.5 Hz)	309.4 ± 64.4	172.3 ± 28.7*	255.3 ± 35.5	150.4 ± 18.5
High frequency (0.5–3 Hz)	456.1 ± 60.8	400.7 ± 69.0	592.5 ± 95.1	456.9 ± 61.2
Total	1,960 ± 324	1,244 ± 176	1,722 ± 229	1,184 ± 126
Cross-spectral parameters (0.3-0.5 Hz)				
Coherence	0.57 ± 0.01	0.57 ± 0.01	0.56 ± 0.01	0.58 ± 0.01
Gain (bpm/mmHg)	12.17 ± 1.32	11.54 ± 0.93	10.69 ± 1.01	9.48 ± 1.14
Phase (pi fraction)	0.05 ± 0.14	0.12 ± 0.14	0.40 ± 0.11	0.17 ± 0.12

In contrast, female GPR37L1^−/−^ mice had changes to spectral power in both MAP and HR spectra during the active period. In the MAP spectra, power in the low and total frequency bands was significantly lower in GPR37L1^−/−^ female mice compared to wildtype (Figures 4E,H). Power in the high and total frequency bands of the HR spectra was also significantly lower in GPR37L1^−/−^ females than wildtype controls (Figures 4K,L; Table 3). During the inactive period, power in HR and MAP spectra within all specified frequency bands was comparable between female GPR37L1^−/−^ and wildtype mice (Table 3). In the segments selected from both the light and dark periods, GPR37L1^−/−^ females had higher MAP than wildtype females (Table 3), concordant with averaged data over the entirety of these respective periods (Table 1; Figures 1A,B).

**Figure 5:**
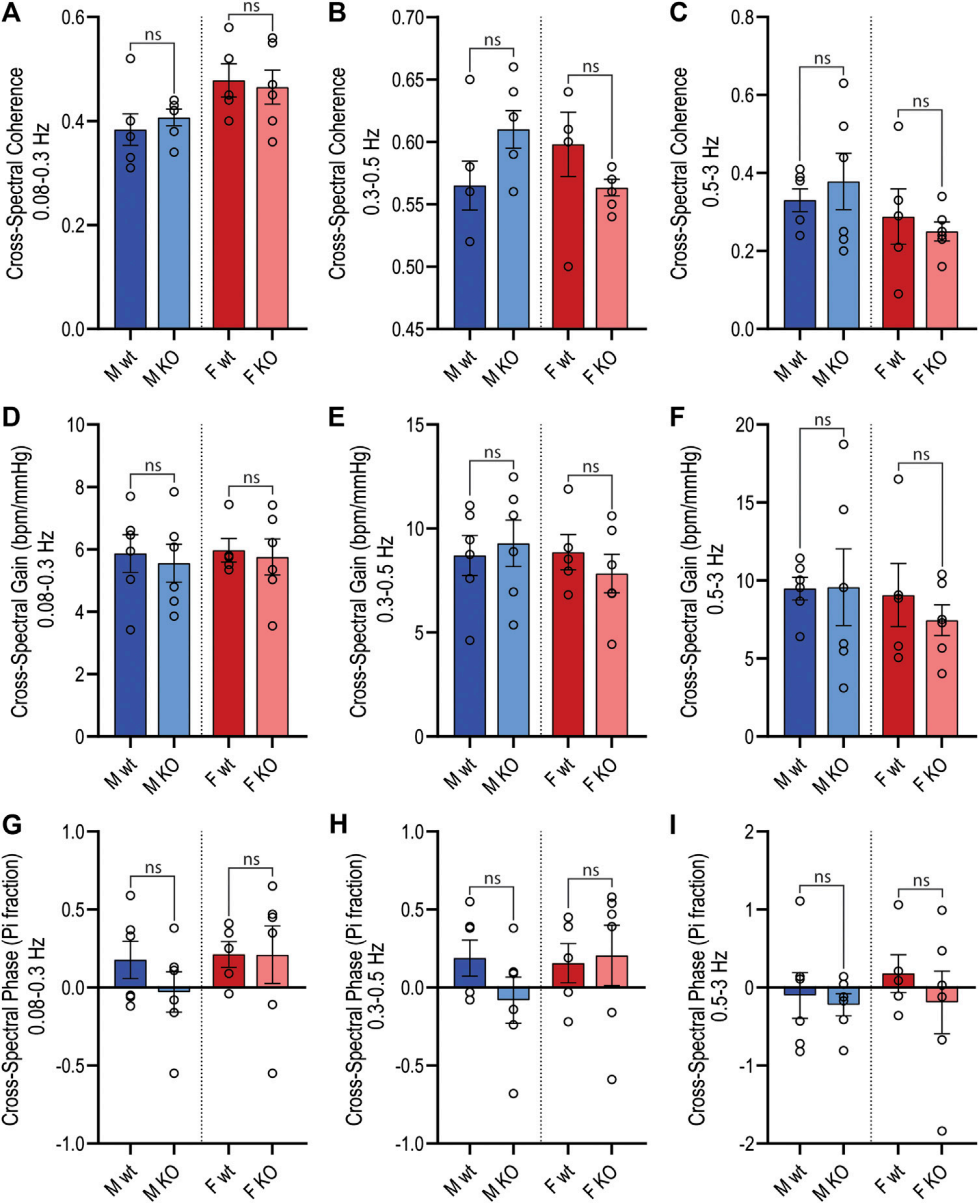
Active period cross-spectral analysis. Cross-spectral parameters for MAP and HR spectra in male and female wildtype (wt, C57BL/6) and GPR37L1^−/−^ (KO) mice derived from radiotelemetry recordings during the active period (1300–0100 h) subjected to cross-spectral power analysis. Spectral coherence (correlation coefficient) between MAP and HR spectra is given in the frequency bands: 0.08–0.3 Hz **(A)**, 0.3–0.5 Hz **(B)** (mid frequency band, associated with SNS fluctuations), and 0.5–3 Hz **(C)**. Cross-spectral gain (change in HR in bpm per mmHg change in MAP) is given in 0.08–0.3 Hz **(D)**, 0.3–0.5 Hz **(E)**, and 0.5–3 Hz **(F)** frequency bands. The phase of HR spectra in relation to MAP spectra is shown in 0.08–0.3 Hz **(G)**, 0.3–0.5 Hz **(H)**, and 0.5–3 Hz **(I)** frequency bands. Males *n* = 6, females *n* = 5–6. Analyzed by split plot ANOVA of four spectral sequences with Bonferroni correction for multiple comparisons. Graphs represent mean ± SEM with individual data points represented by open circles; * represents *p* < 0.05 vs. wt, ns is not significant.

For both sexes, baroreflex sensitivity, estimated by cross-spectral gain between MAP and HR spectra, was comparable between GPR37L1^−/−^ and wildtype mice (Table 3; Figures 5D–F). The relationship coefficient between MAP and HR spectra, given by cross-spectral coherence, was equivalent in GPR37L1^−/−^ and respective sex-matched wildtype mice for both sexes (Figures 5A–C). The temporal relationship between MAP and HR fluctuations is also comparable across genotypes for both sexes (Figures 5G–I).

### Pharmacological Assessment of Sympathetic Contribution to Blood Pressure

**Figure 6:**
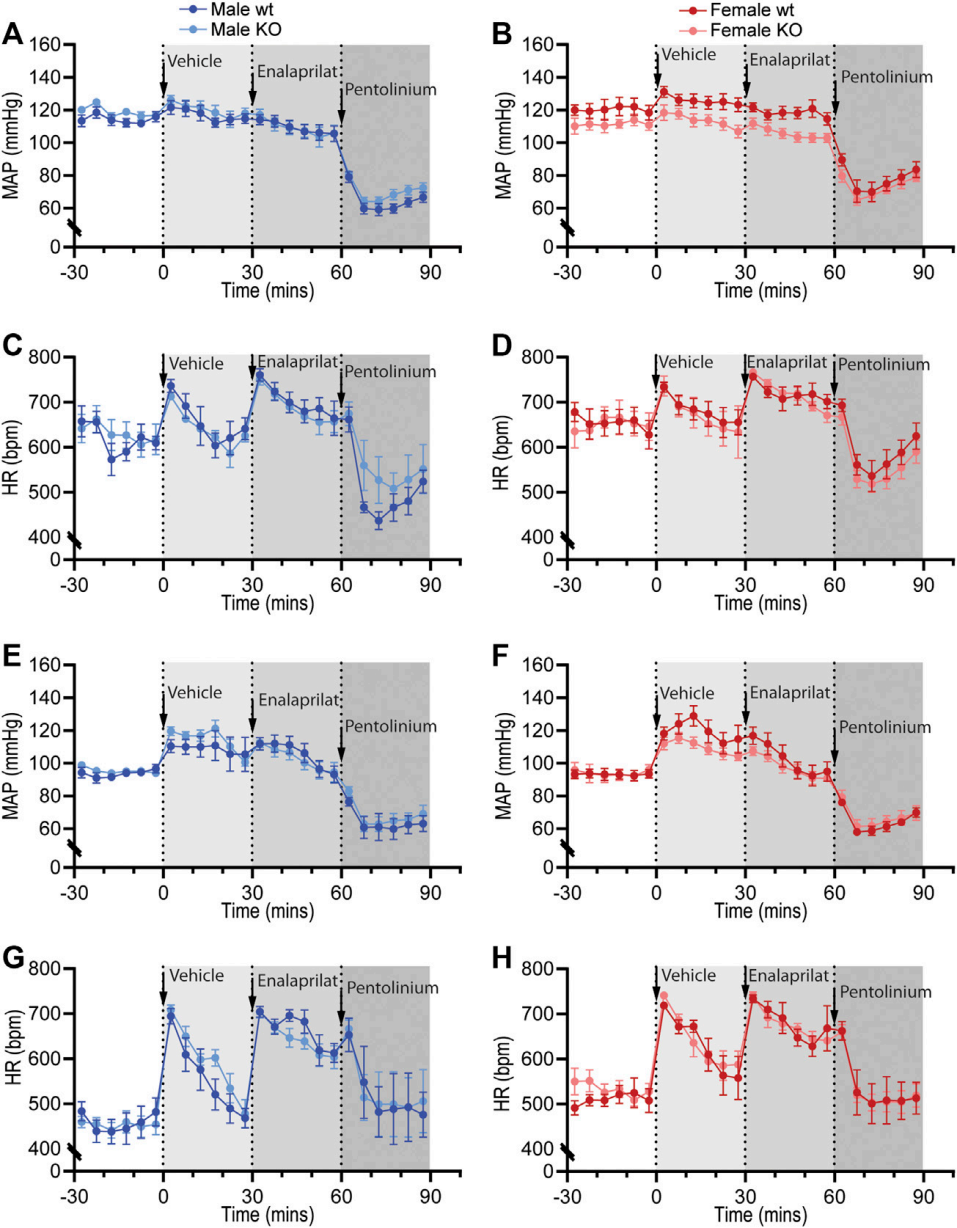
Pharmacological blockade of the renin-angiotensin system and sympathetic nervous system in GPR37L1^−/−^ (KO) mice. Sequential pharmacological blockade of the renin-angiotensin system (enalaprilat, 1 mg/kg IP) and the sympathetic nervous system (pentolinium, 5 mg/kg IP) in 15–17 week old wildtype and GPR37L1^−/−^ mice was performed during the active **(A)–(D)** and inactive **(E)–(H)** periods. MAP was derived from telemetry recordings for both male **(A)**, **(E)** and female mice **(B)**, **(F)**. HR over this period was also recorded for males **(C)**, **(G)** and females **(D)**, **(H)**. Arrows indicate time of IP drug administration. Data points are mean ± SEM for each 5 min interval. Active period male *n* = 6, female *n* = 7; inactive period male *n* = 5–6, female *n* = 6–7.

Sympathetic vasomotor drive was assessed by pharmacological ganglionic blockade with pre-inhibition of the renin-angiotensin system (RAS). RAS inhibition with enalaprilat induced a depressor response and tachycardia in all groups when performed in the active (Figures 6A–D; Table 4) and inactive periods (Figures 6E–H; Table 5). Blood pressure of GPR37L1^−/−^ and wildtype mice responded similarly to enalaprilat treatment during the active period (Table 4); in the inactive period, male GPR37L1^−/−^ mice had a significantly attenuated tachycardia response and female mice showed genotype differences in locomotor activity (Table 5).

**TABLE 4:** Cardiovascular and locomotor responses in GPR37L1^−/−^ and C57BL/6J mice to vehicle, enalaprilat and pentolinium during the active period.

Vehicle																	
	WT male	KO male		WT female		KO female		Effect of sex		Effect of genotype		Genotype X sex		Male WT:KO		Female WT:KO	
ΔSBP (mmHg)	−3.0 ± 1.2	−2.5 ± 1.9		1.2 ± 1.3		−3.5 ± 1.5		>0.50		0.34		0.30		>0.50		0.16	
ΔDBP (mmHg)	−2.4 ± 1.0	−2.4 ± 1.5		1.4 ± 1.4		−2.6 ± 1.4		0.44		0.33		0.38		>0.50		0.19	
ΔMAP (mmHg)	−2.6 ± 1.1	−2.5 ± 1.7		1.3 ± 1.3		−3.1 ± 1.4		0.49		0.31		0.33		>0.50		0.16	
ΔHR (bpm)	−21.6 ± 10.2	−28.0 ± 7.7		−7.8 ± 11.6		−29.1 ± 13.7		>0.50		0.44		>0.50		>0.50		0.40	
ΔActivity (units)	−0.90 ± 0.20	−0.40 ± 0.20		−0.50 ± 0.30		−1.70 ± 0.40		0.30		0.28		0.06		>0.50		0.04	
*n*	6	6		7		7											

Enalaprilat																	
	WT male	KO male		WT female		KO female		Effect of sex		Effect of genotype		Genotype X sex		Male WT:KO		Female WT:KO	
ΔSBP (mmHg)	−8.4 ± 2.3	−12.4 ± 2.1		−6.8 ± 1.7		−7.2 ± 2.7		0.17		0.40		0.46		0.26		>0.50	
ΔDBP (mmHg)	−7.4 ± 2.0	−11.0 ± 1.9		−6.2 ± 1.8		−8.7 ± 2.2		0.43		0.17		>0.50		0.28		0.40	
ΔMAP (mmHg)	−7.5 ± 2.1	−11.4 ± 2.0		−6.4 ± 1.7		−7.6 ± 2.4		0.29		0.29		>0.50		0.26		>0.50	
ΔHR (bpm)	54.8 ± 16.6	48.9 ± 15.0		50.2 ± 10.1		51.1 ± 19.7		>0.50		>0.50		>0.50		>0.50		>0.50	
ΔActivity (units)	0.00 ± 0.40	0.40 ± 0.30		0.10 ± 0.30		0.30 ± 0.50		>0.50		>0.50		>0.50		>0.50		>0.50	
*n*	6	6		7		7											

Pentolinium																	
	WT male		KO male		WT female		KO female		Effect of sex		Effect of genotype		Genotype X sex		Male WT:KO		Female WT:KO
ΔSBP (mmHg)	−44.3 ± 2.8		−36.4 ± 2.6		−43.8 ± 2.1		−30.5 ± 2.6		0.20		<0.001		0.27		0.03		<0.001
ΔDBP (mmHg)	−43.3 ± 2.5		−36.7 ± 1.9		−41.0 ± 2.1		−31.1 ± 2.6		0.08		<0.001		0.46		0.05		0.002
ΔMAP (mmHg)	−45.4 ± 2.7		−37.7 ± 2.1		−43.2 ± 2.1		−31.5 ± 2.6		0.07		<0.001		0.40		0.03		<0.001
ΔHR (bpm)	−215.4 ± 18.4		−139.1 ± 25.5		−149.1 ± 24.3		−159.1 ± 8.3		0.22		0.11		0.02		0.01		>0.50
ΔActivity (units)	−1.00 ± 0.30		−1.10 ± 0.20		−2.10 ± 0.30		−1.50 ± 0.40		0.11		>0.50		0.38		>0.50		0.28
*n*	6		6		7		7										

**TABLE 5:** Cardiovascular and locomotor responses in GPR37L1^−/−^ and C57BL/6J mice to vehicle, enalaprilat and pentolinium during the inactive period.

**Vehicle**																								
	WT male	KO male		WT female			KO female			Effect of sex			Effect of genotype			Genotype X sex			Male WT:KO			Female WT:KO		
ΔSBP (mmHg)	8.1 ± 4.5	9.9 ± 2.4		16.0 ± 2.2			6.7 ± 2.2			>0.50			0.19			0.10			>0.50			0.04		
ΔDBP (mmHg)	7.0 ± 4.5	9.8 ± 2.1		14.0 ± 2.2			6.6 ± 2.1			>0.50			0.36			0.10			>0.50			0.08		
ΔMAP (mmHg)	7.4 ± 4.5	9.3 ± 2.2		15.1 ± 2.2			6.7 ± 2.1			0.49			0.24			0.10			>0.50			0.05		
ΔHR (bpm)	−24.0 ± 23.4	23.0 ± 12.3		9.0 ± 14.0			8.3 ± 11.7			>0.50			0.36			0.30			0.17			>0.50		
ΔActivity (units)	−0.50 ± 0.10	−0.40 ± 0.10		0.60 ± 0.30			−0.60 ± 0.20			0.09			0.003			<0.001			>0.50			<0.001		
*n*	5	6		6			7																	

Enalaprilat																								
	WT male		KO male		WT female			KO female			Effect of sex			Effect of genotype			Genotype X sex			Male WT:KO			Female WT:KO	
ΔSBP (mmHg)	−11.0 ± 2.7		−15.6 ± 3.2		−21.7 ± 2.8			−14.0 ± 2.1			0.22			>0.50			0.06			0.33			0.08	
ΔDBP (mmHg)	−7.3 ± 2.7		−13.0 ± 2.4		−20.3 ± 2.7			−13.9 ± 2.2			0.04			>0.50			0.05			0.21			0.12	
ΔMAP (mmHg)	−8.7 ± 2.7		−13.8 ± 2.7		−21.1 ± 2.8			−13.9 ± 2.2			0.07			>0.50			0.05			0.26			0.09	
ΔHR (bpm)	145.7 ± 15.3		78.6 ± 13.5		71.1 ± 12.4			61.3 ± 17.9			0.06			0.12			0.21			0.05			>0.50	
ΔActivity (units)	0.30 ± 0.10		0.00 ± 0.00		−0.90 ± 0.30			0.00 ± 0.10			0.008			0.05			0.001			0.26			<0.001	
*n*	5		6		6			7																

Pentolinium																								
	WT male		KO male			WT female			KO female			Effect of sex			Effect of genotype			Genotype X sex			Male WT:KO			Female WT:KO
ΔSBP (mmHg)	−37.8 ± 2.1		−30.3 ± 3.3			−32.1 ± 2.5			−27.2 ± 1.4			0.20			0.06			>0.50			0.12			0.27
ΔDBP (mmHg)	−34.2 ± 2.0		−31.9 ± 2.3			−31.8 ± 2.1			−26.5 ± 1.5			0.19			0.20			>0.50			>0.50			0.20
ΔMAP (mmHg)	−37.5 ± 2.0		−32.4 ± 2.8			−33.0 ± 2.3			−27.9 ± 1.5			0.15			0.10			>0.50			0.27			0.23
ΔHR (bpm)	−150.8 ± 27.5		−120.3 ± 33.9			−142.4 ± 15.6			−146.5 ± 18.7			>0.50			>0.50			0.45			0.37			>0.50
ΔActivity (units)	−0.20 ± 0.10		0.30 ± 0.10			0.30 ± 0.10			0.10 ± 0.10			>0.50			>0.50			0.07			0.08			0.45
*n*	5		6			6			7															

Ganglionic blockade with pentolinium elicited a rapid depressor response that was associated with bradycardia in all groups when performed in the active (Table 4) or inactive periods (Table 5). During the active period, the pentolinium-induced reduction of MAP and SBP in both sexes of GPR37L1^−/−^ mice was significantly attenuated compared to their sex-matched wildtype counterparts (Figures 6A,B; Table 4). BP and HR responses to pentolinium during the inactive period were similar between genotypes (Figures 6E–H; Table 4).

### Cardiovascular Reactivity to Aversive and Appetitive Stimuli

**Figure 7:**
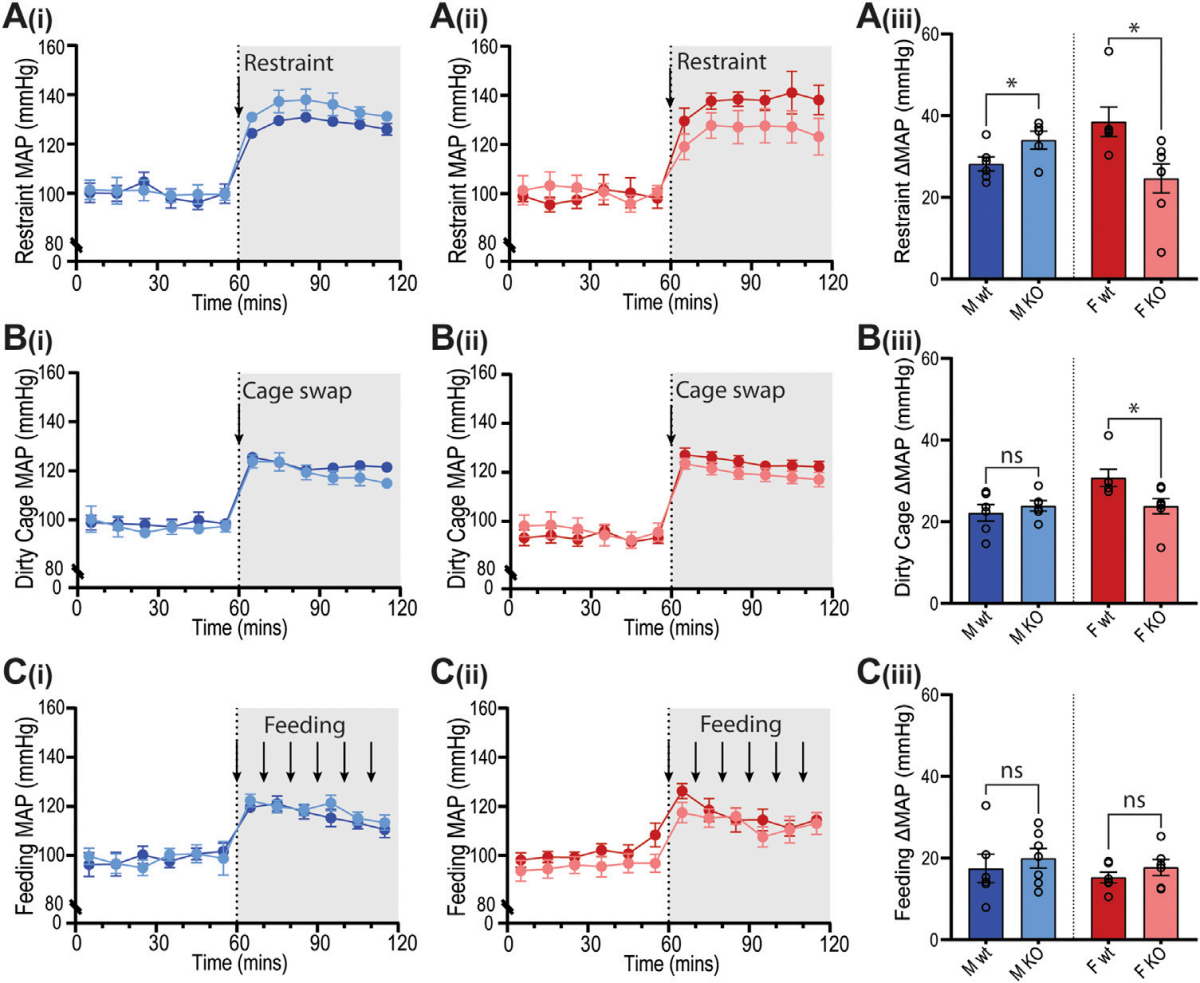
Blood pressure responses of GPR37L1^−/−^ mice to physical stress tests. Wildtype (wt, C57BL/6) and GPR37L1^−/−^ (KO) mice were subjected to a series of stress tests while blood pressure was recorded via radiotelemeter at 14–17 weeks of age. One hour of stable baseline recording was acquired prior to testing; confining mouse inside a plexiglass restraint apparatus (‘restraint’, *n* = 5–6, females *n* = 6–7) **(A)**, swapping mouse into a soiled cage (‘cage swap’, males *n* = 6, females *n* = 6–7) **(B)** and feeding with almond every 10 min (‘feeding’, males *n* = 6–7, females *n* = 6) **(C)**. 10 min averages of mean arterial pressure (MAP) are shown for males **(Ai)**–**(Ci)** and females **(Aii)**–**(Cii)** over the course of each experiment. Change in MAP was determined as the difference between baseline MAP average vs test MAP average **(Aiii)**–**(Ciii)**. XY plot data points are mean ± SEM for 5-min averages. For simplicity, bar graphs show mean ± SEM, with open circles representing average MAP change between control (0–60 min) and test (60–120 min) for individual subjects, though statistical analysis was performed on 5-min averages (XY plot data) using split plot ANOVA with Bonferroni correction for multiple comparisons, * represents *p* < 0.05 vs. wt, ns is not significant.

**Figure 8:**
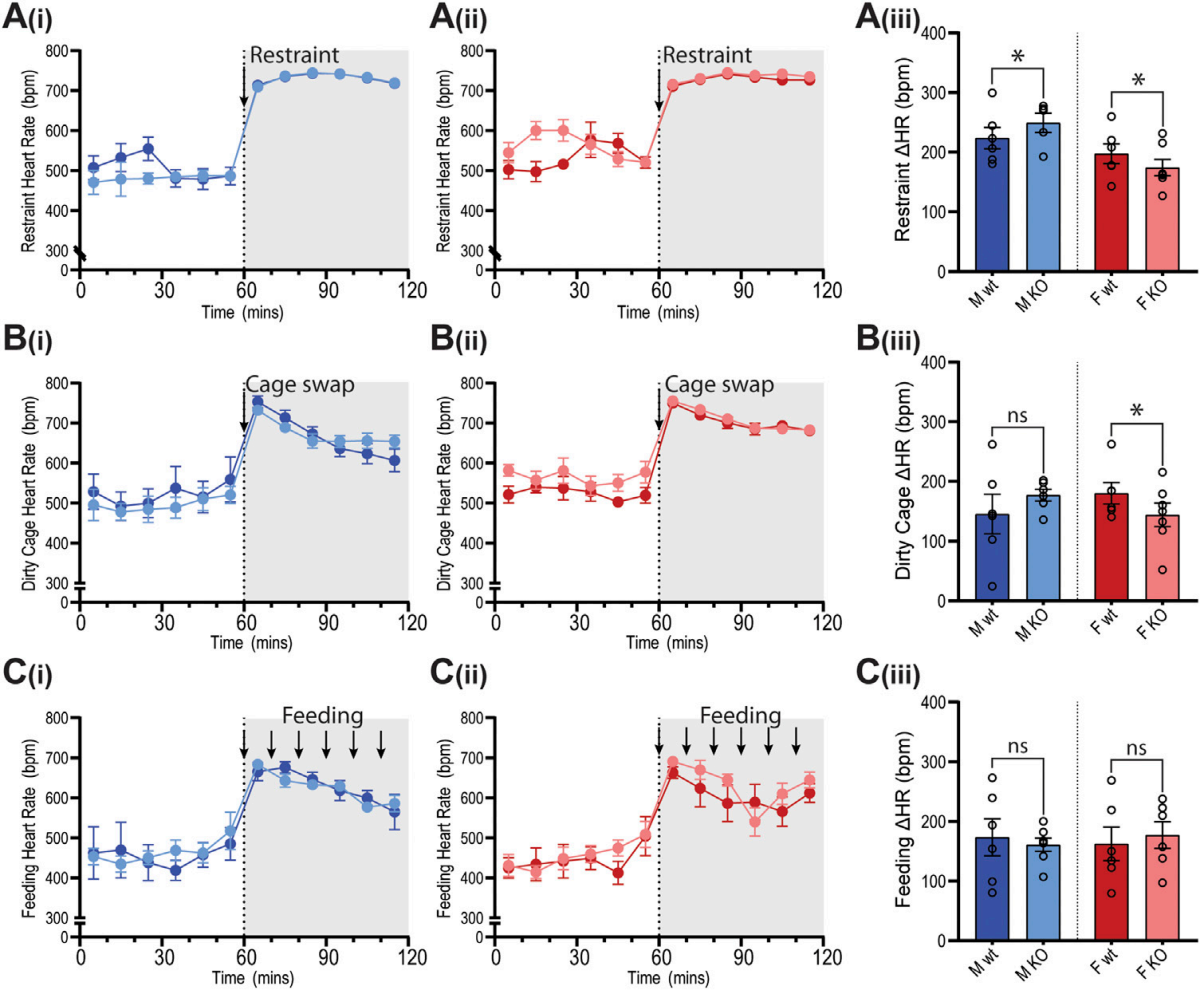
Heart rate of GPR37L1^−/−^ mice in response to physical stress tests. Wildtype (wt, C57BL/6) and GPR37L1^−/−^ (KO) mice were subjected to a series of stress tests while heart rate was recorded via radiotelemeter at 14–17 weeks of age. One hour of stable baseline recording was acquired prior to testing; placing the mouse inside a confined plexiglass restrainer (‘restraint’, *n* = 5-6, females *n* = 6–7) **(A)**, swapping mouse into a soiled cage (‘cage swap’, males *n* = 6, females *n* = 6–7) **(B)** and feeding with almond every 10 min (‘feeding’, males *n* = 6–7, females *n* = 6) **(C)**. 10-min averages of heart rate are shown for males **(Ai)**–**(Ci)** and females **(Aii)**–**(Cii)** over the course of each experiment. Change in heart rate was determined as the difference between baseline heart rate average vs test heart rate average **(Aiii)**–**(Ciii)**. XY plot data points are mean ± SEM for 5-min averages. For simplicity, bar graphs show mean ± SEM, with open circles representing average HR change between control (0–60 min) and test (60–120 min) for individual subjects, though statistical analysis was performed on 5-min averages (XY plot data) using split plot ANOVA with Bonferroni correction for multiple comparisons, * represents *p* < 0.05 vs. wt, ns is not significant.

Cardiovascular reactivity to stress was assessed by subjecting GPR37L1^−/−^ and wildtype mice to physical restraint, dirty cage swap and palatable food presentation (feeding) tests while BP, HR and activity were recorded by telemetry. The three stimuli all elicited immediate pressor responses (Figure 7) and tachycardia (Figure 8) in both GPR37L1^−/−^ and their sex-matched wildtype counterparts.

In GPR37L1^−/−^ female mice, the pressor response to physical restraint (aversive) was significantly attenuated compared to C57BL/6J control females (Figures 7A(ii),(iii)), as was the tachycardic response (Figures 8A(ii),(iii)). In the same test, male GPR37L1^−/−^ mice exhibited a statistically significant augmentation of pressor and tachycardic responses compared to wildtype males (Figures 7A(i),(iii), 8A(i),(iii)). Similarly, pressor response and tachycardia induced by the (aversive) dirty cage swap stimulus was significantly dampened in female GPR37L1^−/−^ mice compared to wildtype females (Figures 7B(ii),(iii), 8B(ii),(iii)). During cage swap, male GPR37L1^−/−^ mice exhibited equivalent increases in blood pressure and HR compared to C57BL/6J males (Figures 7B(i),(iii), 8B(i),(iii)).

In contrast to aversive stressors, the MAP and HR changes induced by an appetitive feeding stimulus in female GPR37L1^−/−^ mice was equivalent to wildtype females, and male GPR37L1^−/−^ mice again responded similarly to their wildtype counterparts (Figures 7C and 8C).

**Figure 9:**
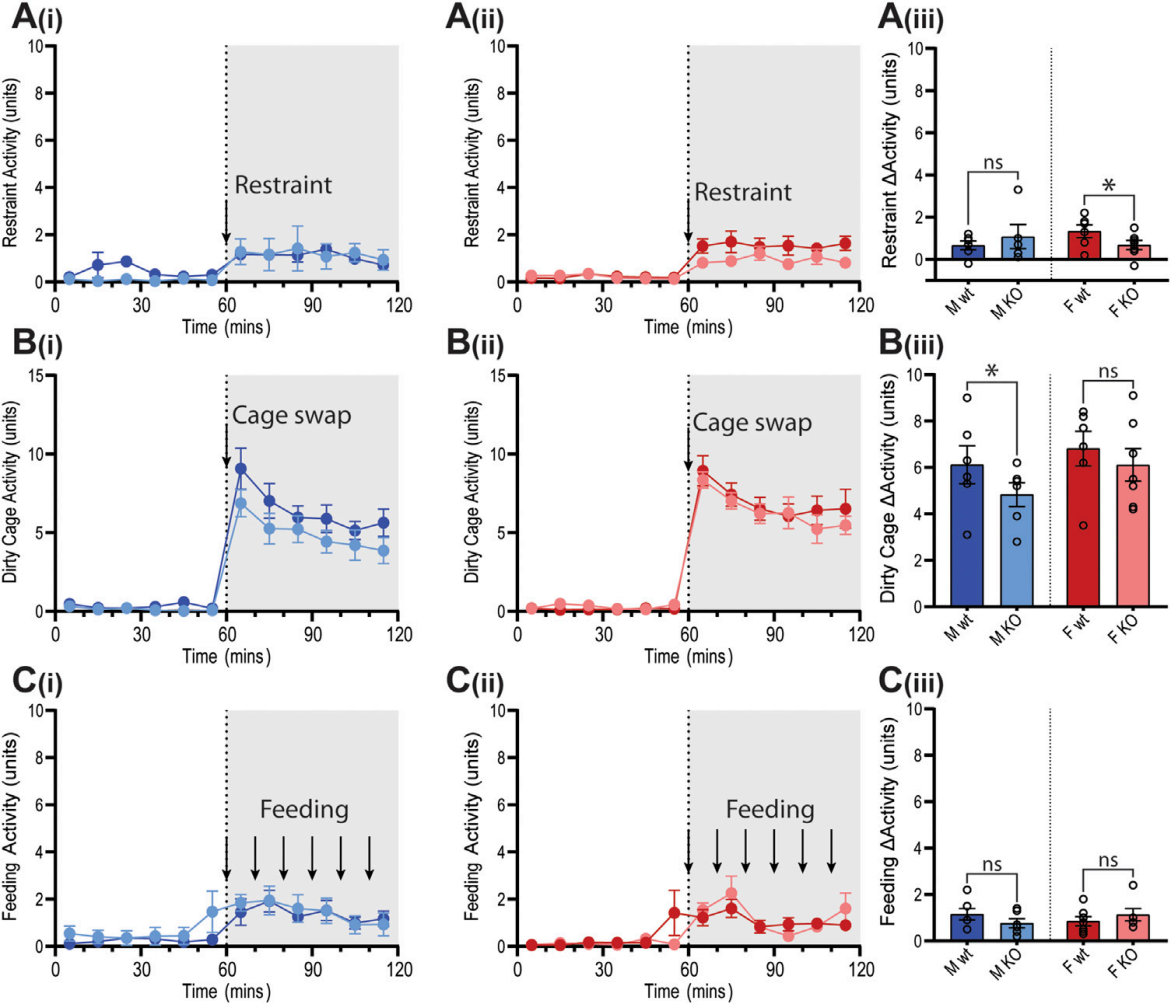
Locomotor responses of GPR37L1^−/−^ mice to physical stress tests. Wildtype (wt, C57BL/6) and GPR37L1^−/−^ (KO) mice were subjected to a series of stress tests while locomotor activity was recorded via radiotelemeter at 14–17 weeks of age. One hour of stable baseline recording was acquired prior to testing; placing the mouse inside a confined plexiglass restrainer (males *n* = 5-6, females *n* = 6–7) **(A)**, swapping mouse into a soiled cage (males *n* = 6, females *n* = 6–7) **(B)** and feeding with almond every 10 min (males *n* = 6-7, females *n* = 6) **(C)**. 10-min averages of locomotor activity are shown for males **(Ai, Bi, Ci)** and females **(Aii, Bii, Cii)** over the course of each experiment. Change in activity was determined by the average activity during test compared to the average of the baseline period for each test **(Aiii, Biii, Ciii)**. XY plot data points are mean ± SEM for 5-min averages. For simplicity, bar graphs show mean ± SEM, with open circles representing average activity change between control (0–60 min) and test (60–120 min) for individual subjects, though statistical analysis was performed on 5-min averages (XY plot data) using split plot ANOVA with Bonferroni correction for multiple comparisons, * represents *p* < 0.05 vs. wt, ns is not significant.

All three tests induced increases in locomotor activity (Figure 9). Stimulus-induced elevation in locomotor activity was significantly attenuated in female GPR37L1^−/−^ mice during restraint test (Figure 9A), and in male GPR37L1^−/−^ during the dirty cage stress (Figure 9B). This female-specific attenuation of the pressor response in GPR37L1^−/−^ mice following aversive, but not appetitive, stresses was independent of locomotor activity, as restraint and dirty cage tests induce vastly different increases in activity level (Figure 9).

### Light-Dark Transition Test for Basal Anxiety

**Figure 10:**
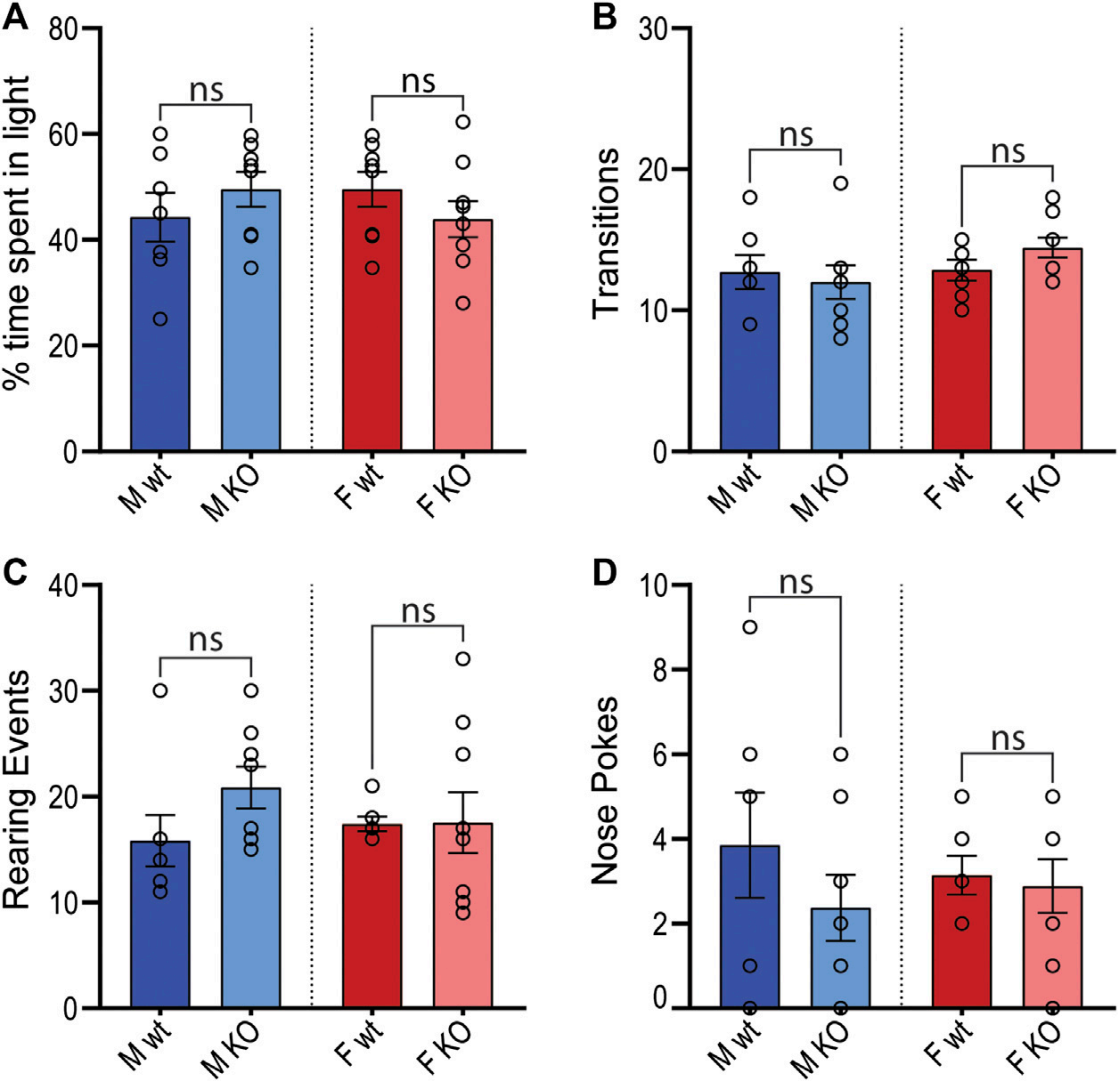
Light-dark transition anxiety test. Wildtype (wt, C57BL/6) and GPR37L1^−/−^ (KO) mice were placed into the light-dark test apparatus and parameters indicative of anxiety behaviors were scored; percentage of time spent in the light section of apparatus **(A)**, number of transitions between sections **(B)**, number of rearing events **(C)** and number of nose pokes not followed by complete transition **(D)**. Males *n* = 7–8, females *n* = 7–9. Analyzed by ordinary two-way ANOVA with Bonferroni correction for multiple comparisons. Graphs represent mean ± SEM with open circles representing individual subjects, * represents *p* < 0.05 vs. wt, ns is not significant.

To determine whether GPR37L1^−/−^ mice had chronic anxiety-related behavior that may also influence blood pressure, mice were subjected to the light-dark transition test. GPR37L1^−/−^ mice of both sexes showed no statistical difference in the percentage of time spent in the light zone, or in the number of transitions between light and dark zones (Figures 10A,B). Measures of exploratory behavior (rearing events, nose pokes) were also comparable between GPR37L1^−/−^ and wildtype mice of either sex (Figures 10C,D).

## Discussion

The orphan GPCR, GPR37L1, has a proposed role in brain development and function (Marazziti et al., 2013) but more recently has been suggested to influence blood pressure (Min et al., 2010; Coleman et al., 2018; Zheng et al., 2018). The present study used mice with genetic deletion of *Gpr37L1* to investigate how this receptor contributes to cardiovascular regulation. This involved assessing short term cardiovascular variability, sympathetic vasomotor tone and baroreflex mechanisms as well as behavioral and cardiovascular responses to aversive and non-aversive stress. We found that loss of GPR37L1 increased heart rate and reduced sympathetic vasomotor tone in mice of both sexes, while female mice lacking GPR37L1 had reductions in blood pressure, cardiac vagal drive, and cardiovascular reactivity to aversive stimuli compared to wildtype females.

To establish basal blood pressure, heart rate and locomotor activity, radiotelemetry was performed in mice with global deletion of *Gpr37L1* (GPR37L1^−/−^) and wildtype control mice from the same line (C57BL/6J background). Mice of both sexes were included in the study as it has been previously shown that deletion of *Gpr37L1* has sexually dimorphic effects in mice (Coleman et al., 2018). It is becoming increasingly obvious that males and females utilize different integrated physiological mechanisms to buffer and maintain cardiovascular homeostasis (Hart et al., 2009; Mouat et al., 2018), thus it is important to investigate how mechanisms may differ between the sexes. The mechanism by which GPR37L1 effects change in the cardiovascular system is unclear, though we propose it interacts either directly or indirectly with central cardiovascular control regions. GPR37L1 is expressed in glial cells throughout the brain, though most highly in the cerebellum (Valdenaire et al., 1998; Lein et al., 2007). It is known that the cerebellar vermis influences cardiovascular activity by both sympathoinhibitory and sympathoexcitatory functions mediated by Purkinje cell clusters (Paton and Spyer, 1992), and that the cerebellum forms connections with the hypothalamus (Cavdar et al., 2001) and with the nucleus of the solitary tract (Somana and Walberg, 1979). These are important areas of central cardiovascular control that are known to be affected by estrogen (Hay et al., 2014), which may partially account for the sex differences seen in the cardiovascular phenotype of GPR37L1^−/−^ mice. Importantly, expression of GPR37L1 itself does not appear to differ between males and females (Coleman et al., 2018), thus the sexually dimorphic cardiovascular phenotype seen in GPR37L1^−/−^ mice likely arises secondarily due to innate sex differences in cardiovascular control, though we cannot exclude the possibility of male-female differences in receptor activation.

Similar to our previous study examining cardiovascular parameters in this line (Coleman et al., 2018), we observed that blood pressure of male GPR37L1^−/−^ mice did not differ from wildtype males, with good agreement in blood pressure values for males between the studies. However, while our previous report observed higher blood pressure in GPR37L1^−/−^ females vs. wildtype controls (Coleman et al., 2018), here we found lower blood pressure in female GPR37L1^−/−^ mice compared to their wildtype counterparts. When we directly compared cardiovascular parameters between the studies, we observed statistically higher SBP of wildtype female mice in the present study compared to that of Coleman et al. (2018) during the active period. Further, wildtype females are the only group in this study that do not have lower inactive period SBP (attributable to lower heart rate in the recordings in this study) than their counterparts in the Coleman et al. (2018) study. This analysis was done by two-way ANOVA, with a Bonferroni post-hoc test (data not shown). It is clear that the explanation lies in the blood pressure difference of female wildtype mice across the studies, rather than in the female GPR37L1^−/−^ mice. The blood pressure of wildtype female mice used here and previously (Coleman et al., 2018) is within normal biological variation of MAP for C57BL/6 mice reported in studies using radiotelemetry (Tiemann et al., 2003; Xue et al., 2005; Nohara et al., 2013; Sabharwal et al., 2016; Bruder-Nascimento et al., 2017), thus the differences seen between the present study and Coleman et al. (2018) may be attributable to chance selection of experimental groups, or potentially environmental factors that were not standardized between research sites (Richter et al., 2009).

We were interested to determine whether the differences in blood pressure in female GPR37L1^−/−^ mice compared to wildtype were driven by the differences in locomotor activity, as exercise is associated with both an increase in sympathetic activity and an increase in blood pressure (Nobrega et al., 2014). To address this, we investigated the correlation between MAP and locomotor activity (as log-transformed activity levels). There were no statistical differences in the regression slope or correlation coefficient of GPR37L1^−/−^ mice compared to wildtype, indicating that the magnitude of the MAP-activity relationship remains intact in the knockout mice. GPR37L1^−/−^ female mice had a significantly offset regression, with lower average MAP than wildtype females at 0 log activity, indicating that the blood pressure differences between genotypes of female mice is independent of locomotor activity level.

After basal cardiovascular monitoring, the telemetry recordings of heart rate and blood pressure were subjected to power spectral analysis to investigate whether short-term cardiovascular fluctuations are altered in GPR37L1^−/−^ mice, which may indicate changes in autonomic activity. Notably, we did not see a significant decrease in spectral power within the 0.3–0.5 Hz frequency band in MAP, which centers around the resonance frequency of the baroreceptor reflex, and is an indicator of sympathetic tone (Janssen and Smits, 2002). Despite this, there was significant attenuation of MAP fluctuations in female GPR37L1^−/−^ mice compared to wildtype in the low (0.08–0.3 Hz) and total (0.08–10 Hz) frequency bands, which may represent changes in other cardiovascular homeostatic mechanisms. We also observed significant attenuation of HR power in the high frequency range (0.5–3 Hz) in female GPR37L1^−/−^ mice during the active period, fluctuations in which are primarily parasympathetically mediated (Gross et al., 2005; Baudrie et al., 2007). Considering this, we suggest that cardiac vagal input is lessened in these mice, leading to disinhibition of the cardiac pacemaker and thus explaining the higher heart rate in GPR37L1^−/−^ mice (Table 1). Indeed, the higher heart rate in the knockout mice is within the range that is achieved by inhibition of parasympathetic activity (Gross et al., 2005) or concomitant inhibition of sympathetic and parasympathetic systems (Åstrand et al., 2004). Additionally, male GPR37L1^−/−^ mice had lower power in the low and mid (0.3–0.5 Hz) frequency bands of HR spectra during the inactive period. These differences represent trends toward dampening of short-term rhythmic fluctuations in both HR and MAP for both sexes of GPR37L1^−/−^ mice across the frequencies measured, manifested more prominently in females. We detected no change to the cardiovagal arm of the baroreflex in GPR37L1^−/−^ mice by assessment of cross-spectral gain between HR and MAP in the frequency domain (Table 3), indicating that these changes to short-term fluctuations in MAP and HR are not associated with aberrant cardiovagal baroreflex function.

As there is a decrease in spectral power in GPR37L1^−/−^ that is not localized to a specific frequency band, there may be broader changes in autonomic activity and other cardiovascular regulatory mechanisms than initially expected. As has been observed, broader dampening of heart rate variability can occur as a result of either high or low states of autonomic activity (Scheff et al., 2014). Thus, it is not possible to infer the level of autonomic activity in GPR37L1^−/−^ mice from power spectral analysis alone.

As such, we additionally investigated sympathetic influence on the cardiovascular system by an alternative method; mice were subjected to autonomic blockade with pentolinium, a common method for assessing sympathetic control of vasomotor tone (Young and Davisson, 2011). During the active period, we observed that GPR37L1^−/−^ mice of both sexes have attenuated depressor responses to pentolinium (Table 4). As blood pressure in mice is predominantly determined by sympathetic tone (Janssen et al., 2000), this attenuated reduction in blood pressure following pentolinium administration is likely due to lower sympathetic vasomotor tone in GPR37L1^−/−^ mice.

We note that while ganglionic blockade and power spectral analyses showed an association between GPR37L1 and autonomic regulation of blood pressure, these are indirect measures of autonomic nervous system activity and not without limitation (Young and Davisson, 2011). Indeed, power spectral analysis is not capable of detecting changes in autonomic outflow to particular vascular beds, and in some cases, elevated sympathetic nerve activity (as determined by direct electrode measurement) is not associated with changes in power in the sympathetic-associated range of blood pressure frequency spectra (Stauss et al., 1995). Direct measures of renal sympathetic nerve activity in conscious mice have only been performed in a single study (Hamza and Hall, 2012), and are not capable of detecting baseline differences between genotypes, thus they are beyond the scope of the present work. The results presented here provide evidence for a link between GPR37L1 and autonomic blood pressure control, which warrants further investigation by more comprehensive methods that directly assess sympathetic and parasympathetic activity.

The sympathetic nervous system is also involved in cardiovascular arousal following environmental stress (Curtis and O’Keefe, 2002). As such, we sought to investigate whether GPR37L1^−/−^ mice have altered cardiovascular responses to stress. We determined that there are no differences in basal anxiety levels in GPR37L1^−/−^ mice of either sex compared to wildtype controls, as indicated by the light-dark box test. This is concordant with an independent GPR37L1 knockout strain showing normal exploratory behavior at 3 months of age (Jolly et al., 2018). GPR37L1^−/−^ and wildtype mice of both sexes were also exposed to a series of acute stressors, which produced prompt and sustained increases in MAP and HR for mice of both genotypes, the magnitudes of which are in line with previous studies (Jackson et al., 2007; Chen et al., 2009; Abegaz et al., 2013). Interestingly, when presented with aversive and appetitive stimuli, GPR37L1^−/−^ female mice showed cardiovascular reactivity that was dependent on the emotional valence of the stimulus; attenuated blood pressure and heart rate elevation to restraint and dirty cage stress, and normal cardiovascular response to the innocuous feeding stimulus. A similar phenomenon is seen in renin enhancer knockout mice, which exhibit a reduced cardiovascular response to aversive but not appetitive stimuli, as well as slightly lower basal blood pressure compared to their wildtype counterparts (Jackson et al., 2007), and lower power in mid frequency MAP spectra and low and mid frequency HR spectra (Adams et al., 2006). It is known that cardiovascular reactivity to both aversive and non-aversive stressors is sympathetically mediated (Randall et al., 1985; Carrive, 2002), which eliminates the possibility that GPR37L1^−/−^ mice simply have either dampened sympathetic outflow or decreased responsiveness to sympathetic nerve activity, given that female GPR37L1^−/−^ mice mount a normal cardiovascular response to appetitive stimuli, and that male GPR37L1^−/−^ mice have augmented responsiveness to restraint. Rather, these stimuli-dependent cardiovascular responses may be due to abnormal stimulus assessment or fear response by the medial amygdala (Davern and Head, 2011).

We also cannot exclude that cardiovascular homeostatic mechanisms other than autonomic activity are involved in this phenotype. It is known that sex has effects on many buffering mechanisms including the renin-angiotensin system (Mouat et al., 2018) and nitric oxide synthase (McNeill et al., 1999), which may also contribute to the sex differences observed in GPR37L1^−/−^ mice. We also have not investigated directly whether there are perturbations to the relative contributions of sympathetic and parasympathetic systems to cardiovascular homeostasis in GPR37L1^−/−^ mice, though considering the findings presented here, we believe further investigation is warranted.

At a cellular level, the molecular effects of GPR37L1 activation are still poorly understood. As GPR37L1 is specifically expressed in astrocytes, it seems plausible that this receptor may play a role in modulating glutamatergic signaling by affecting expression or function of astrocytic glutamate transporters. However, global deletion of GPR37L1 does not affect mRNA abundance of key murine astrocytic glutamate transporters GLT-1 and GLAST in the hippocampus of mice at 14 or 30 days of age (Jolly et al., 2018). Furthermore, electrophysiology in hippocampal slice culture also revealed that D-aspartate induced currents in astrocytes were comparable between wildtype and *Gpr37l1* global knockout mice, and kainite- and AMPA-induced currents in C1 pyramidal neurons were also the same between genotypes (Jolly et al., 2018), suggesting that loss of GPR37L1 does not affect neuronal glutamate signaling at least in the hippocampal region of *Gpr37l1* knockout mice. Alternatively, it has been suggested that GPR37L1 associates with the dopamine D_2_ receptor (D_2_) in cells, as analyzed by fluorescence cross-correlation spectroscopy (Hertz et al., 2018), though a negative control was not used in these experiments, and a direct interaction between GPR37L1 and the D_2_ receptor has not been shown.

In summary, we have shown that GPR37L1^−/−^ mice exhibit reduced sympathetic vasomotor tone in both males and females, possibly due to changes in autonomic activity, and show that the perturbed cardiovascular responses to stressful stimuli are sexually dimorphic. The exact mechanism by which GPR37L1 exerts these effects is yet to be determined, though it is clear this receptor has a centrally mediated role in maintaining cardiovascular homeostasis.

## Data Availability

The raw data supporting the conclusions of this article will be made available by the authors, without undue reservation.
